# Maxillary odontogenic myxoma involving the maxillary sinus - Case report

**DOI:** 10.1016/S1808-8694(15)30586-3

**Published:** 2015-10-19

**Authors:** Allan Ulisses Carvalho de Melo, Sérgio Bartolomeu de Farias Martorelli, Paulo Henrique de Holanda Cavalcanti, Luiz Alcino Gueiros, Fernando de Oliveira Martorelli

**Affiliations:** 1 Specialist in Public Health and Master in Buccal Diagnosis - UFPB. Dentist – Oncology Department - Hospital Gov. João Alves Filho.; 2 Specialist and Master in Maxillo-Facial Surgery and PhD in Stomatology - UFPB. Full Professor of Maxillo-Facial Surgery and Traumatology – Dentistry School of Recife, FOR.; 3 Specialist and Master in Maxillo-Facial Surgery and Traumatology – Adjunct Professor of Maxillo-Facial Surgery and Traumatology – Dentistry School of Recife, FOR.; 4 Stomatology and MSc. In Bucall Diagnosis - UFPB. Substitute Professor of Stomatology - UFPE.; 5 Dentistry Student – Caruaru dental School Dental School of Recife and Federal University of Paraiba Faculdade de Odontologia de Recife e Universidade Federal da Paraíba.

**Keywords:** myxoma, maxillary sinus, odontogenic tumors

## Abstract

The aim of this paper is to report a case of odontogenic myxoma that affected the right maxilla and maxillary sinus. We have also reviewed the literature in regards of the clinical, radiographic, histological and treatment aspects of this pathology. Odontogenic myxomas of the maxillofacial region are benign lesions, without preference for gender, race or location, with extremely varied clinical and radiographic characteristics, thus increasing the number oral and maxillofacial region tumors with which we can make the differential diagnosis.

## INTRODUCTION

Myxoma is a benign, slow-growth, mesenchimal-stemed and locally aggressive neoplasia. Virchow coined this term in 1863, because he believed that, as it happens with the umbilical chord, this disease had mucin. Myxomas may involve hard and soft tissue (heart, subcutaneous, skin, and others). Usually, when it involves bony tissue, it affects the facial bones^1^, ^2^, ^3^.

The odontogenic myxoma is a rare, benign tumor that does not shed metastasis and involves the maxillo-mandibular complex. When involving the maxilla, odontogenic myxomas can expand to inside the maxillary sinus, and are then diagnosed later only after having grown to larger sizes. They may still involve the palate, orbit and nasal cavity, causing symptoms associated with these structures^1^, ^3^, ^4^.

The odontogenic nature of the maxillary myxomas stemming from a tooth germ may be proven through the following facts: it rarely occurs in other bones if not on the face; it has a histological similarity with the dental mesenchyma; it is associated with unerupted or absent teeth; and there is a sporadic presence of epithelial islands or odontogenic tissue inside the myxomatous stroma^2^, ^3^, ^4^, ^5^.

## LITERATURE REVIEW

According to the latest WHO's classification of odontogenic tumors, in 1992, the myxoma is considered a tumor of the odontogenic mesenchyma, with or without the presence of odontogenic epithelium. The rarity of this pathology may be seen in some publications, such as in a retrospective study of 30 years, carried out at the Oral Pathology Laboratory of the Dentistry School of the Federal University of Rio Grande do Norte, where they analyzed 127 cases of odontogenic tumors, and the odontogenic myxoma corresponded to 4.72% of the sample with 3 cases seen in men and in women alike^6^, ^7^.

Odontogenic maxillary myxomas were first mentioned in the literature by Thoma and Goldman in 1947. They usually affect adolescents and young adults, between the second and third decades of life, very rarely affecting people before 10 years of age or after 50 years of age. There is no consensus in the literature regarding location predilection (maxilla or mandible) and gender (males or females), however, most researchers state that there is no racial predilection^1^, ^8^.

Just as it happens to most odontogenic tumors, the odontogenic myxomas are asymptomatic, causing pain, paresthesia or asymmetries only when they take on larger sizes. Their growth is usually slow, however they are locally aggressive. They may cause divergence or root resorption, tooth shifting or movement^2^, ^4^, ^8^.

In a recent prospective study involving 33 cases of odontogenic myxomas, the most affected individuals were within the age range of 10 and 39 years, and they affected mostly the posterior portion of the mandible. Since most cases were advanced, the most common symptoms were pain and cortical bone perforation with invasion of soft tissue. The most commonly found radiographic characteristics were multilocularity and root resorption^5^.

Although the radiographic aspects of odontogenic myxomas are markedly variable, they are always radiolucent. They may present with the aspect of soap bubbles, tennis racket and honey combs; there have been reports of a sun ray aspect 3, 9.

In a retrospective radiographic analysis of 21 cases, the mandible was more involved than the maxilla. We also noticed that the unilocular forms were usually located in the anterior maxilla, while the multilocular forms involved the posterior region^9^.

Both conventional radiographs and CT scans must be used in the radiographic investigation in order to size the tumor, define its margins, establish bony septum aspects and investigate whether or not there is cortical perforation^10^.

In its histological aspect, the odontogenic myxoma has abundant mucosal intercellular substance, made up of eosinophilic lax connective tissue, immersed in this stroma there are spindle-like cells and star-shaped cells with elongated cytoplasm, with or without small masses of inactive odontogenic epithelium. We have seen recently myxoma cases with characteristics that had not been described before in the literature, among them we have the presence of round calcified bodies made up of bony-cement tissue and islands of active odontogenic epithelium^11^, ^12^.

Histologically, differential diagnosis must be made with rabdomyosarcoma, myxoid liposarcoma, neurogenic sarcoma, neurofibroma, lipoma, fibroma, chondromyxoid and nodular faciitis^8^.

Clinical and radiographic differential diagnosis of odontogenic myxomas may include: intraosseous hemangioma, cherubism, aneurysmatic bony cyst, fibrous dysplasia, ameloblastoma, gigantic cells central lesion, traumatic bony cyst and odontogenic cysts (radicular, lateral periodontal, dentigerous and keratocyst)^2^, ^8^, ^9^.

Since odontogenic myxomas bear a high risk of recurrence, mainly due to its gelatinous aspect and having no capsule, it is necessary that the initial treatment be very efficient. One important aspect that should be taken into account is the possibility that the patients may skip control visits. Thus, a possible recurrence will be seen only later on and treatment will be impaired. For all these reasons, a resection with broad margins is the most indicated treatment. 5, 10.

Post-operative proservation of patients with odontogenic myxomas is indefinite, especially in the first two years, period of the greatest recurrence rate.^4^

## CLINICAL CASE PRESENTATION

Female, Caucasian, 23 years old, coming from Recife-PE, came to our attention complaining of a bulging in her right maxillary region.

**Figure 1:**
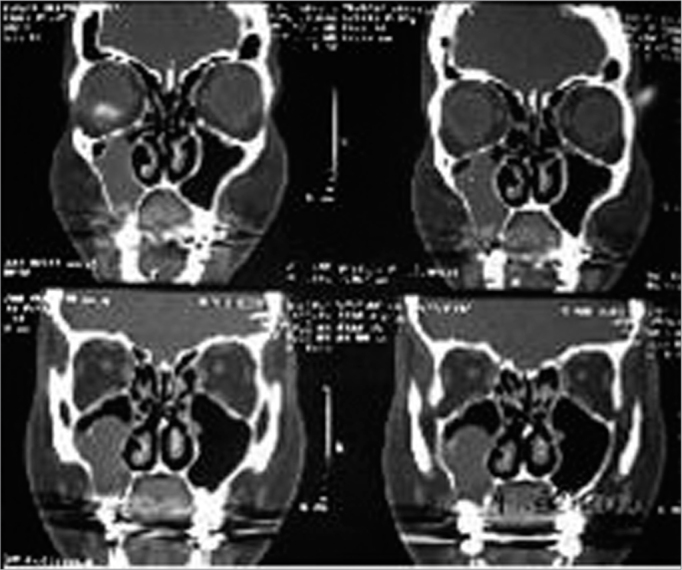
Coronal view CT scan showing a tumor invading the right maxillary sinus.

During clinical exam we noticed an enlargement in that region where tooth 16 had been removed (right side first molar) 12 months before. The overlaying mucosa was normal. Panoramic x-ray of the maxillary bone and periapical x-ray of the area showed an osteolithic lesion in the edentulous region and root resorption in the regions of the 15th (2nd right superior pre-molar) and 17th (2nd right superior molar) teeth. Other preoperative exams were normal. Medical history, family history and life style were uneventful. We then carried out an incisional biopsy under local anesthesia and the histology showed it to be odontogenic myxoma. After being aware of such diagnosis, we ordered a CT scan with a window for hard and soft tissue, axial and coronal views, which showed a lesion invading the right maxillary sinus, extending throughout the entire wall of the maxillary sinus, extending all the way to the orbit floor (Figure 1).

**Figure 2:**
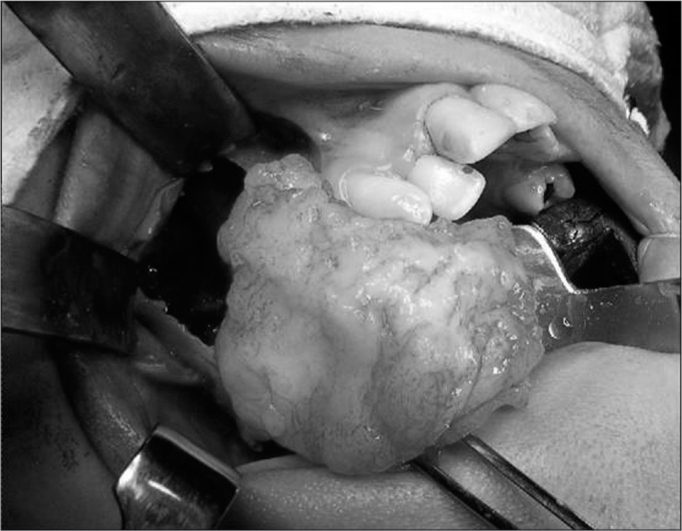
Transoperative view showing bone resection to remove a maxillary odontogenic myxoma.

**Figure 3:**
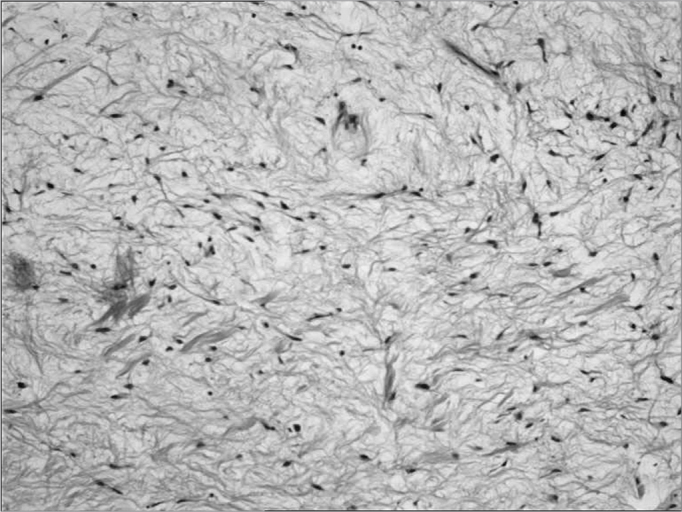
Histopathological aspect of the maxillary odontogenic myxoma, dyed in HE, 100X.

New preoperative exams were ordered and they were all within normal parameters. We then operated her under inhaling anesthesia and nasotracheal intubation. Through a modified Neuman incision we exposed the anterior wall of the maxillary sinus and cut the bone, removing the entire tumor together with teeth numbers 15, 17 and 18, which were affected by the lesion that measured 5cm in its largest diameter (Figure 2). Histopathology of the surgical specimen confirmed the diagnosis of odontogenic myxoma, because of the presence of spindle-like cells, with egg-shaped or elongated nuclei, without cell atypia, immerse in loose and myxomatous matrix, including scarce blood vessels and irregular calcification sites (Figure 3).

In the 6th month of postoperative control we did not find clinical or radiographic signs of recurrence. This case will be under indefinite surveillance.

## DISCUSSION

We have reported a case of odontogenic myxoma involving the maxilla and the maxillary sinus. This disease's slow but aggressive growth rate, makes it reach large sizes and, when involving the maxilla, it can invade the entire maxillary sinus, as was the case here^1^, ^3^, ^4^.

Since we are unable to assign specific clinical and radiographic characteristics to odontogenic myxomas, all lesions that may be considered as such must undergo incisional biopsy, as it happened in our case. Thus, we can avoid transoperative surprises and a possible incomplete tumor removal^13^.

Because of the large size of the tumor hereby reported and the slow growth of myxomas, one can suspect that such tumor was already present when the first right superior molar was removed because of a carious lesion. Initial radiographic alterations in the bony tissues can pass unnoticed if a detailed analysis of the panoramic radiographies is not carefully done.

Cases like this also show the need to order panoramic radiographs within an annual dental check-up, and also periapical x-rays in situations that require greater details. CT scans can be ordered whenever it is necessary to see detailed characteristics of the lesion associated with its size, internal configuration, and others^14^.

Clinical and radiographic characteristics of the odontogenic myxoma are variable, thus it should be always considered in the differential diagnosis of mixed and radiolucent lesions in maxillas of all age ranges. One less common clinical characteristic found in our case was root resorption^15^.

Since it is an infiltrative, aggressive disease, with a high recurrence rate, treatment performed in this case hereby reported was bony resection, because besides allowing entire tumor enucleating, it removes bone tissue that could be microscopically involved, and then cause disease recurrence^3^.

## FINAL COMMENTS

Clinical and radiological aspects of maxillary odontogenic myxomas are not conclusive, it is necessary to have a histopathological exam for the final diagnosis. Because of its high rate of recurrence, especially due to its gelatinous and mucous aspect, surgical treatment through bone resection is the most indicated treatment modality, and the patient must be followed up closely for years.
